# Place of residence and childhood cancer survival

**DOI:** 10.18632/oncotarget.26717

**Published:** 2019-03-08

**Authors:** Arash Delavar, Kimberly J. Johnson

**Affiliations:** Brown School Master of Public Health Program, Washington University in St. Louis, St. Louis, MO, USA; Siteman Cancer Center, Washington University in St. Louis, St. Louis, MO, USA

**Keywords:** rural, residence, pediatric, cancer, survival

Cancer treatment in the United States is a complicated endeavor, often requiring a team of experts including physicians, psychologists, child-life specialists, and social workers [1]. Depending on the type and severity of the cancer, as well as one's location and access to care, treatment may be given through a Comprehensive Cancer Center, which is designation given by the National Cancer Institute for centers with resources available to conduct translational research and clinical trials [2]. These centers have the ability to treat many different cancer types. There are 49 National Cancer Institute-designated comprehensive cancer centers in 36 states, often located in large metropolitan areas [2]. Due to logistical issues that might be associated with accessing these resources, one might expect inferior survival for patients living in rural areas compared to urban areas.

However, this does not appear to be the case for childhood and adolescent cancer patients. Our study examining childhood and adolescent cancer survival in the United States found no variation by rural/urban residence at the time of diagnosis as defined by rural-urban continuum codes (RUCCs) [3], in contrast to previously published studies on US adults [4], and children abroad [5]. To understand why, it is worth speculating on how rural children and adolescents in the United States experience cancer.

Children and adolescents are more likely to be insured than their adult counterparts [6], which is partially attributable to extended eligibility through the Children's Health Insurance Program [7]. Because insurance access can vary by place of residence, uniform coverage may lessen any survival disparities between rural and urban populations. Even when a family must relocate closer to a hospital so their child can get treatment – a burden disproportionately faced by rural childhood and adolescent cancer patients and their families [8] – public and private assistance is available to help pay for the cost of travel and lodging [9]. Though we can always do more to make life for childhood cancer patients and their families easier, it is worth noting that rural adult cancer patients may not have the same resources available to them.

Beyond access, children and adolescents have the added benefit of parental or guardian surveillance and support during the course of their illness. An observant parent may quickly notice changes in their child's health and take action, whereas a single adult in a rural area may delay seeking medical attention. Even if resources are available, rural adults have been reported to be less receptive to accessing healthcare resources, as well as being more likely to exhibit healthcare avoidant behaviors [10]. Further, because health behaviors likely play a larger role in the incidence and treatment of adult cancers compared to childhood cancers, behavioral risk factors and exposures associated with being in a rural setting may disproportionately affect adults.

One result that was not the focus of our study is particularly noteworthy and merits further discussion. When we stratified our analysis by race, we observed worse survival for rural American Indian/Alaskan Native children and adolescents compared to those in urban areas (Hazard Ratio: 2.24; 95% Confidence Interval: 1.10-4.56) [3]. Although this finding was incidental and should be considered exploratory due to a small sample size (281 cases) [3], it is possible that factors unique to some members of this population, such as living on an Indian reservation, may result in worse access to care for those in rural areas [11]. Other known challenges for American Indian/Alaskan Native children and adolescents, such as relatively high rates of child maltreatment, low socioeconomic status, a poorly funded healthcare system (Indian Health Service), and high rates of medical mistrust among adults could contribute to disparities if they are exasperated in rural populations [11, 12].

It is possible that elements inherent to the experience of childhood cancer, such as parental or guardian surveillance and support, may explain why rural children and adolescents overall do not show worse survival than their urban counterparts. However, future studies could explore other factors – especially those that may be shaped by policy – that protect certain populations from rural/urban health disparities. These factors could theoretically guide policies that seek to lessen rural/ urban health disparities for other groups. As cancer care continues to grow more complex and specialized, efforts should be made to prevent all populations living in rural communities from encountering difficulties in the pursuit of effective cancer care.
